# When robot knocks, knowledge locks: how and when does AI awareness affect employee knowledge hiding?

**DOI:** 10.3389/fpsyg.2025.1627999

**Published:** 2025-07-18

**Authors:** Zhen Liu, Quanxing Lin, Shimin Tu, Xin Xu

**Affiliations:** ^1^School of Management, Shanghai University, Shanghai, China; ^2^School of Journalism and Communication, Shanghai University, Shanghai, China

**Keywords:** AI awareness, knowledge hiding, psychological availability, person-organization fit, conservation of resource theory, social identity theory

## Abstract

**Introduction:**

Artificial intelligence (AI) technology is fundamentally reshaping organizational knowledge management practices. This study explores the mechanism between AI awareness and knowledge hiding, focusing on psychological availability and person-organization fit as key mediating and moderating variables. The research provides valuable insights into the psychological drivers of knowledge hiding behavior under AI-induced stress, contributing to a deeper understanding of employee counterproductive behaviors in the context of technological change.

**Method:**

We surveyed 311 employees from various industries in China, analyzing the data using SPSS 27.0, PROCESS 4.0, and AMOS 29.0, and employed structural equation modeling (SEM) to examine the relationships among AI cognition, psychological availability, person-organization fit, and knowledge hiding. The study tested the mediating effect of psychological availability between AI cognition and knowledge hiding, as well as the moderating role of person-organization fit in this process.

**Results:**

AI awareness is positively correlated with knowledge hiding behavior, with psychological availability playing a partial mediating role. Additionally, person-organization fit moderates the relationship between AI awareness and psychological availability. A stronger fit between employees and the organization weakens the negative impact of AI awareness on psychological availability, thereby reducing knowledge hiding behavior.

**Discussion:**

This study is the first to empirically test how psychological availability links AI awareness with employees' knowledge hiding behavior, enriching the theoretical understanding in the field of knowledge hiding. The research also highlights the importance of person-organization fit in mitigating the negative effects of technological change. By integrating the Conservation of Resources Theory and Social Identity Theory, this study offers practical recommendations for organizations managing knowledge sharing challenges in AI-driven environments.

## 1 Introduction

Artificial Intelligence (AI) technologies, as the core engine of the so-called Fourth Industrial Revolution, are fundamentally transforming organizations' decision-making processes, business operations, structural configurations, and knowledge management paradigms (Glikson and Woolley, 2020; Haefner et al., 2021; Mahmud et al., 2022; Vrontis et al., 2022). In the context of an accelerating knowledge economy, knowledge has undoubtedly become a pivotal strategic resource for sustaining competitive advantage (Santhose and Lawrence, 2023). Knowledge sharing and collaborative cooperation among organizational members serve as vital drivers for organizational innovation and enhanced performance outcomes (Xu and Wei, 2023). However, knowledge hiding, a typical form of counterproductive work behavior, functions as an invisible barrier that obstructs the diffusion and integration of knowledge within organizations. In this study, knowledge hiding is defined as “an intentional attempt by an individual to withhold or conceal knowledge that has been requested by another person” (Connelly et al., 2012). This behavior can induce a breakdown of trust among employees, reduce team creativity and collaborative efficiency, and ultimately erode core organizational competitiveness by deteriorating cultural cohesion and weakening innovation capacity (He et al., 2021; Siachou et al., 2021). These risks become particularly salient in environments where AI technologies are increasingly embedded into organizational workflows. The uncertainty and threat appraisals triggered by such technological transitions may further amplify employees' propensity to engage in knowledge hiding. Yet, the mechanisms underlying this relationship remain insufficiently theorized and empirically tested (Arias-Perez and Velez-Jaramillo, 2022; Kim and Kim, 2024). Although prior scholarship has extensively examined the antecedents of knowledge hiding under traditional organizational contexts, including personality traits, leadership styles, and organizational climate (Shen et al., 2025), there is limited empirical attention given to how AI-related cognitive factors, especially AI awareness, influence knowledge hiding behaviors within technologically volatile settings.

It has been projected by the World Economic Forum that by 2025 AI will be capable of performing 52 percent of tasks within enterprises, thereby reducing the share of tasks executed by employees to 48 percent (Arias-Pérez and Huynh, 2023). Such a shift is intensifying occupational anxiety among manufacturing employees and is poised to permeate knowledge intensive industries (Kim and Kim, 2024). In this study, AI awareness is defined as employees' threat perception regarding the potential for AI technologies to replace their job roles or undermine their occupational value, which, at its core, constitutes an individual's subjective appraisal of the risk of resource loss (Brougham and Haar, 2018; Mo et al., 2024; Hobfoll, 1989).

According to the Conservation of Resources (COR) theory, when individuals perceive a threat to their resources, defensive motivations are activated, leading them to adopt strategies aimed at preserving resource balance (Hobfoll, 1989; Hobfoll et al., 2018). Knowledge, as a core occupational resource for employees, can be deliberately hidden to avoid dilution of one's competitive advantage in the workplace through knowledge sharing. This enables employees to maintain their irreplaceability amid AI-driven technological transformations (Connelly et al., 2012; Cerne et al., 2014). Li et al. (2019) found that the implementation of AI technologies exacerbates employees' concerns regarding their career development.

Arias-Perez and Velez-Jaramillo (2022) contend that under technological turbulence, employees tend to protect their personal interests through knowledge hiding. Accordingly, AI awareness constitutes a critical antecedent that triggers employees' knowledge hiding behaviors. To gain a deeper understanding of how AI awareness influences knowledge hiding, this study further examines psychological availability as a mediating mechanism in this relationship. Based on the Conservation of Resources theory, psychological availability was originally conceptualized by organizational behavior scholar Kahn in 1990 and is defined herein as an individual's subjective perception of possessing physiological, emotional, and cognitive resources, reflecting their motivational state (Kahn, 1990). Specifically, this motivational state encompasses two dimensions: having the capability to perform and possessing the motivation to perform (Qian et al., 2020). Prior research has demonstrated that stress and job insecurity significantly reduce employees' psychological availability (Restubog et al., 2011; Wang et al., 2021). Given that AI awareness functions as a persistent stressor, it is highly likely to decrease psychological availability through these pathways. Therefore, it is posited that AI awareness negatively affects psychological availability. Moreover, the level of psychological availability directly influences employees' behavioral choices (Connelly et al., 2012; Cerne et al., 2014). The study by Qian et al. (2020) also confirmed a positive association between psychological availability and knowledge sharing behaviors. Hence, it is reasonable to infer that psychological availability may serve as a mediator in the relationship between AI awareness and knowledge hiding.

The complex relationships among these variables often depend on the interaction between individual characteristics and organizational context (Zhu et al., 2022). To uncover the boundary conditions under which AI awareness influences knowledge hiding, this study draws upon the Social Identity Theory (SIT) and introduces person–organization fit as a moderating variable. Person–organization fit is defined as the similarity and compatibility between an individual and an organization in terms of values, goals, and characteristics (Kristof, 1996), and its effectiveness hinges on the congruence of values, interests, beliefs, and needs between the two parties (Cable and Edwards, 2004). Social Identity Theory posits that when individuals strongly identify with their organization, they incorporate organizational interests into their self-concept (Tajfel, 1979) and are more inclined to regulate their behavior from the organization's perspective (Ashforth and Mael, 1989). Therefore, person–organization fit is expected to shape employees' behavioral logic by strengthening organizational identification (Kristof, 1996), thereby fostering positive personal attitudes and behaviors (Shalley et al., 2000). Prior research indicates that employees with high person–organization fit are more likely to engage in discretionary behaviors that benefit colleagues and the organization (Zhu et al., 2022). Such employees tend to internalize organizational norms (Xiao et al., 2018), proactively engage in altruistic behaviors, and view knowledge sharing as a role obligation (Brickson, 2013). This identification mechanism enables them to maintain cooperative tendencies even in the face of external threats, since a sense of belonging motivates individuals to prioritize organizational welfare and encourages active participation in organizational activities (Ashforth and Mael, 1989). Moreover, during the organizational knowledge management process, person–organization fit continually exerts a transmitting effect by linking the individual to organizational climate and values, thereby helping the organization overcome challenges (Erdogan et al., 2020; Erdogan and Bauer, 2009). Accordingly, it is reasonable to hypothesize that person–organization fit moderate the primary drivers of knowledge hiding. However, empirical research on the moderating effect of person–organization fit on knowledge hiding behavior remains extremely limited, suggesting that this study addresses a significant gap in the literature.

In summary, this study primarily addresses the following research questions. First, does AI awareness influence employees' knowledge hiding behavior, and if so, through what mechanisms? Second, does psychological availability mediate the relationship between AI awareness and employees' knowledge hiding behavior? Finally, does person–organization fit moderate the mediating effect of psychological availability on the relationship between AI awareness and knowledge hiding?

This study makes four primary contributions. First, by integrating the Conservation of Resources Theory and the Social Identity Theory in the context of AI driven technological transformations, it elucidates the internal mechanism whereby AI awareness influences knowledge hiding through psychological availability, thereby enriching the literature on counterproductive behaviors under technological change. Second, this research constitutes the first empirical test of the mediating role of psychological availability in the relationship between AI awareness and knowledge hiding, deepening understanding of the transmission mechanism between these constructs. Third, by revealing the moderating effect of person–organization fit on the aforementioned pathway, this study not only extends the boundary conditions of knowledge hiding behaviors but also broadens the application of Social Identity Theory to human–machine collaboration contexts. Finally, this work offers both theoretical and practical guidance for managers, assisting them in formulating effective strategies to mitigate the negative impact of employee knowledge hiding.

## 2 Theoretical framework and development of hypotheses

### 2.1 AI awareness and knowledge hiding

In the field of organizational behavior, AI awareness is regarded as a key construct driving employee knowledge hiding behavior (Brougham and Haar, 2018; Mo et al., 2024). The unpredictability of the external environment often triggers opportunistic behavioral patterns in individuals, leading employees to make decisions based on the principle of maximizing their own interests. Specifically, alongside the rapid iteration and widespread application of AI technologies, the technological environment within organizations is undergoing continuous dynamic change, significantly threatening employees' job security. Against this backdrop, knowledge hiding emerges as an important strategy for employees to safeguard their own interests (Arias-Perez and Velez-Jaramillo, 2022). During collaboration with AI, when employees perceive limitations on their employment prospects or the inability to realize their professional value, they experience a psychological sense of resource deprivation, which may be alleviated through knowledge hiding behaviors (He et al., 2024). Moreover, even in the absence of legal ownership, individuals may develop a subjective perception of “psychological ownership” over certain entities, which substantively influence their behavioral expressions. Specifically, algorithmic management enhances employees' psychological ownership of personal knowledge, thereby indirectly promoting the occurrence of knowledge hiding behaviors (Liu et al., 2025).

Based on the Conservation of Resources Theory (COR), anything perceived by individuals as valuable can be regarded as a resource. Individuals constantly strive to acquire and maintain resources they deem valuable, including material resources, psychological resources, condition resources, and personal resources (Hobfoll, 1989). When individuals perceive that their existing resources are at risk of loss or insufficient to meet demands, a strong motivation to protect these resources is activated, prompting defensive behaviors aimed at preserving resource balance (Hobfoll, 1989). Within the context of AI technology deeply embedded in organizational operations, employees face unprecedented challenges in maintaining resource equilibrium. The rapid advancement of AI technology is likely interpreted by employees as a significant threat to critical personal resources such as job security, skill value, and career development (Rampersad, 2020). This subjective perception that AI may replace their jobs or diminish their professional value is referred to as “AI awareness” (Brougham and Haar, 2018; Mo et al., 2024), which essentially reflects employees' negative appraisal of the risk of resource loss induced by AI technologies (Mo et al., 2024).

As AI technology is increasingly deployed across various business functions and processes within organizations, employees' levels of AI awareness are likely to rise significantly (Rampersad, 2020). Existing research indicates that this negative perception triggers individual stress and job insecurity, often leading to deeper anxieties about the undervaluation of one's self-worth (Li et al., 2019). Knowledge is widely recognized as a critical resource for employees in the workplace; when employees perceive AI as a threat that may replace them, their individual resource protection mechanisms are activated to safeguard the self. In such circumstances, employees tend to adopt proactive defensive strategies to mitigate the potential risk of resource loss (Lingmont and Alexiou, 2020). Knowledge hiding, an important defensive tactic in knowledge management, is defined as an employee's deliberate concealment or withholding of relevant information when colleagues explicitly request knowledge. It primarily manifests in three forms: evasive hiding (e.g., providing false information or promising to share later), playing dumb (e.g., feigning ignorance or inability), and rationalized hiding (e.g., refusing to share citing confidentiality rules) (Connelly et al., 2012). When employees perceive that AI may replace their positions or undermine their professional uniqueness, their resource preservation motivation is triggered, leading them to engage in defensive behaviors to reduce potential losses (Lingmont and Alexiou, 2020). As a proactive defensive strategy, knowledge hiding allows employees to monopolize key knowledge and limit others' competitive advantages by reducing knowledge sharing, thereby enhancing their own irreplaceability within the organization (Kim and Kim, 2024). Therefore, we hypothesize that AI awareness positively influences employees' knowledge hiding behavior.

H1: AI awareness is positively related to knowledge hiding.

### 2.2 The mediating role of psychological availability

The Conservation of Resources (COR) Theory proposes that individuals are fundamentally driven to acquire, protect, and maintain valuable resources, including time, energy, and social support. It further states that the perceived threat of losing these resources prompts defensive behaviors aimed at conserving remaining ones (Hobfoll, 1989). Common forms of threat appraisal, including work stress and job insecurity, have been shown to significantly deplete individuals' physiological, emotional, and cognitive resources, thereby reducing their level of psychological availability (Restubog et al., 2011; Wang et al., 2021). Psychological availability was defined by Kahn (1990) as an individual's subjective perception of possessing the physiological, emotional, and cognitive resources necessary for work. Its core dimensions are “having the capability to perform” and “possessing the motivation to perform” (May et al., 2004; Qian et al., 2020). As a critical reflection of motivational state, psychological availability directly influences individuals' behavioral choices (Cerne et al., 2014).

Previous research has demonstrated that psychological availability significantly facilitates knowledge sharing behavior. When employees exhibit high levels of psychological availability, the dimension of “having the capability to perform” manifests as their self-efficacy regarding knowledge sharing, while the dimension of “possessing the motivation to perform” translates into the intrinsic drive to engage in sharing behaviors. Together, these dimensions jointly promote knowledge sharing (Qian et al., 2020). Conversely, when psychological availability is low, employees are more likely to exhibit defensive rather than constructive responses. Under conditions of perceived scarcity of psychological resources, individuals tend to adopt resource-protective strategies in their behavioral decisions. Knowledge hiding, defined as employees' intentional concealment or withholding of knowledge to avoid sharing (Connelly et al., 2012), enables individuals to preserve core occupational resources by preventing dilution of their competitive advantage through sharing, thereby maintaining their irreplaceability and job security (Cerne et al., 2014; Connelly et al., 2019; Halbesleben et al., 2014). Accordingly, reduced psychological availability positively predicts knowledge hiding behavior because it undermines the motivational and capability foundations necessary for engaging in knowledge sharing. Employees with low psychological availability are more prone to feelings of exhaustion and anxiety, making it difficult to meet the additional cognitive and emotional demands required for knowledge sharing (Amabile et al., 2005; Shalley et al., 2004). Empirical evidence confirms that psychological availability sharply decreases under conditions of insecurity or stressful events, as resources are reallocated toward defensive mechanisms (Danner-Vlaardingerbroek et al., 2013; Li and Tan, 2013).

Building on the rapid technological iteration prevailing today, the development of artificial intelligence (AI) has been recognized as a salient stressor in the employee work environment. AI Awareness is defined as employees' perception and concern that AI technologies may replace or alter their job roles and diminish their occupational value. When such a threat is perceived by employees, negative emotional responses, namely anxiety, self-doubt, and job insecurity, is triggered (Carlson and Frone, 2003; Kahn, 1990). The core impact of threat perception lies in two respects: on one hand, the efficiency of AI technologies may undermine employees' identification with their work value; on the other hand, job content and role uncertainty introduced by AI technologies tend to lower employees' preparedness and confidence in coping with change (Wang et al., 2021). These adverse psychological states are significantly depleting of individuals' physiological, emotional, and cognitive resources (Karahanna and Straub, 1999; Wang et al., 2021). According to the Resource Conservation Theory, the anticipation of resource loss inherent in expected AI substitution itself constitutes a threat, which further precipitates defensive resource protection tendencies (Hobfoll, 1989; Karahanna and Straub, 1999). Ultimately, employees' perception of their resource availability, or Psychological Availability, is diminished through a resource depletion pathway whereby sustained stressors are exerted (Kahn, 1990; Wang et al., 2021). In summary, it is posited that psychological availability mediates the relationship between AI awareness and knowledge hiding.

H2: Psychological availability mediates the relationship between AI awareness and knowledge hiding.

Psychological availability, as a foundational concept in organizational behavior, was first introduced by Kahn (1990). May et al. (2004) defined it as the degree to which individuals are ready to deploy their physical, emotional, and cognitive resources when engaging in their work roles. The core of this construct lies in a dynamic self-assessment of whether these internal resources are sufficient to meet task demands. Such assessment encompasses not only individuals' perceptions of their current resource reserves but also their anticipation of potential factors that may hinder task engagement (Cai et al., 2018).

Existing research has extensively examined the antecedents of psychological availability. At the individual level, both the objective level of resource reserves and their subjective evaluations jointly shape employees' psychological availability. High self-efficacy, positive affective states, and physical wellbeing have been shown to significantly enhance individuals' perceived accessibility of personal resources (Kahn, 1990). In addition, individuals' perceptions of entitlement, such as job autonomy and participation in decision-making, may indirectly improve psychological availability by strengthening employees' sense of control (Wang and Yu, 2022). At the leadership level, leadership styles play a particularly salient role. Empowering leadership, by delegating decision-making authority, enhances employees' perceived control over resources (Wang H. et al., 2024). Inclusive leadership, on the other hand, fosters a sense of psychological safety, thereby reducing emotional resource depletion (Guan, 2016). Research on the outcomes of psychological availability has also been substantial. Kahn (1990) demonstrated that employees with high levels of psychological availability are more likely to enter a state of deep work engagement. Binyamin and Carmeli (2010) found that psychological availability positively predicts employee creativity. Under empowering leadership, it facilitates the generation of innovative ideas by reducing employees' perceptions of risk. Wang and Yu (2022) further revealed that employees with higher psychological availability are more inclined to challenge organizational routines, which in turn promotes deviant innovation behaviors.

In the era of artificial intelligence, rapid technological advancements have significantly enhanced employee work efficiency. However, they also provoke anxiety and job insecurity among employees who perceive that artificial intelligence may replace their roles. This perception is referred to as artificial intelligence awareness (AI awareness). The stress, insecurity, and interference associated with AI awareness deplete employees' resources and reduce their psychological availability (Restubog et al., 2011). Consequently, employees become concerned about their resource status, which undermines their belief in accessing physical, emotional, and cognitive resources (Wang et al., 2021).

From the perspective of physiological resources, anxiety triggered by employees' perception that artificial intelligence may replace their jobs activates the sympathetic nervous system, which tends to place individuals in a prolonged state of vigilance and defense (Bjorntorp, 2001). This chronic stress response is primarily manifested at the neuroendocrine level by a sustained increase in cortisol levels, which subsequently leads to dysregulation of the autonomic nervous system (Russell and Lightman, 2019). The depletion of physiological resources includes not only direct physical fatigue but also a decline in restorative capacity. When individuals allocate substantial energy to defensive monitoring induced by AI awareness, the secretion cycle of melatonin, responsible for bodily repair during sleep, may be disrupted (Russell and Lightman, 2019). The chronic stress state elicited by AI awareness continuously consumes physiological functioning, ultimately resulting in insufficient physiological resource reserves that hinder effective engagement in work roles (Avey et al., 2009, 2010, 2008).

Kahn (1990) noted that emotional resources depend on a foundation of psychological safety and meaningfulness. From the perspective of emotional resources, uncertainty regarding technological substitution can trigger emotional exhaustion (Lee and Ashforth, 1996). When employees perceive that artificial intelligence technology may threaten their professional identity, existential anxiety arises. This emotional impact is more destructive than ordinary work stressors (Byrne et al., 2017). Negative career development expectations triggered by AI awareness weaken employees' job security, while a relative deprivation of the perceived value of their skills undermines their sense of meaningful work. Taken together, AI awareness damages the emotional resource base of employees, leading to emotional resource depletion.

From the perspective of cognitive resources, when individuals face threats of resource loss, they experience stress responses and reallocate limited resources to cope with such threats (Hobfoll, 1989). The perceived threat associated with AI awareness is not a momentary event but rather a persistent stressor that requires employees to continuously invest cognitive resources in assessing the risk of being replaced. This ongoing cognitive load encroaches upon the cognitive resources that should otherwise be devoted to work roles (Abiemo et al., 2024).

In summary, we contend that AI awareness leads to the depletion of employees' physiological, emotional, and cognitive resources. Given that these three types of resources constitute the core components of psychological availability (May et al., 2004), such depletion directly diminishes individuals' readiness to evaluate whether their resources are sufficient to meet task demands, namely psychological availability. Therefore, we propose that AI awareness negatively affects psychological availability.

H3: AI awareness is negatively related to psychological availability.

Knowledge hiding refers to the deliberate concealment or withholding of knowledge by individuals when requested by colleagues, manifesting primarily in evasive hiding, feigning ignorance, and rationalized hiding (Connelly et al., 2012). Existing research indicates that individual cognitive and motivational factors are critical triggers for knowledge hiding behaviors (Sun et al., 2022). Psychological availability, as an important cognitive-motivational mechanism, measures the readiness of individuals' resources across physiological, emotional, and cognitive dimensions. Its essence lies in individuals' dynamic evaluation and efficacy perception of their available resource stocks (May et al., 2004). With the deep integration of artificial intelligence technology in modern organizations, employees face increasingly complex challenges, making such self-assessments of resource availability more determinative in their knowledge strategy choices.

According to the Conservation of Resources theory (Hobfoll, 1989), when individuals perceive that they possess abundant resources, a “resource gain spiral” effect can emerge whereby resource accumulation strengthens their confidence in coping with challenges, thereby promoting proactive resource investment behaviors such as knowledge sharing. Conversely, a perceived scarcity of resources tends to trigger defensive resource protection strategies, including knowledge hiding.

From the perspective of physiological resources, employees with higher psychological availability can effectively regulate physiological stress responses triggered by work stressors such as AI applications, thereby maintaining autonomic nervous system balance (Russell and Lightman, 2019). Specifically, when employees perceive that they have sufficient physical capacity to meet demands, their prefrontal cortex can effectively inhibit amygdala-driven defensive responses (Bjorntorp, 2001). In contrast, individuals with lower psychological availability tend to experience prolonged elevated cortisol stress levels, and hyperactivation of the limbic system easily triggers a “fight-or-flight” defensive mode. Such dysregulation of physiological mechanisms significantly increases their propensity to engage in knowledge hiding behaviors (Avey et al., 2009, 2010).

From the perspective of emotional resources, when individuals perceive that their emotional resources are sufficient to cope with external environmental changes, including uncertainties brought by artificial intelligence, they tend to regard proactive knowledge sharing as a positive strategy to strengthen organizational relationships rather than a threat to job security (Cabrera et al., 2006). This positive emotional resource cycle motivates employees with high psychological availability to consolidate and enhance their status within organizational networks through knowledge sharing (Serenko and Bontis, 2016). Conversely, individuals experiencing emotional resource depletion due to sustained anxiety are more likely to view knowledge hiding as a necessary means to protect their occupational standing (Connelly et al., 2012, 2019; Connelly and Zweig, 2015).

From the perspective of cognitive resources, high psychological availability indicates that individuals possess sufficient cognitive resources to process external pressures and information, such as understanding colleagues' knowledge requests. Such employees are better able to clearly evaluate the value of knowledge requests and thus tend to choose knowledge sharing rather than hiding. In contrast, individuals with insufficient cognitive resources often perceive knowledge requests as additional cognitive burdens and tend to adopt knowledge hiding as a coping strategy to reduce cognitive load (Hernaus et al., 2019; Huo et al., 2016; Jha and Varkkey, 2018).

In summary, a high level of psychological availability reflects employees' abundant resources in physiological energy regulation, emotional security, and cognitive flexibility. The effective integration of these multidimensional resources substantially enhances employees' confidence in coping with external challenges, such as AI-driven changes, thereby motivating proactive resource investment behaviors like knowledge sharing rather than defensive resource protection strategies such as knowledge hiding. Consequently, individuals with higher psychological availability are better equipped to mobilize the necessary resources to meet work demands, resulting in a lower tendency to engage in knowledge hiding. Based on the above analysis, this study posits that psychological availability has a significant negative effect on employees' knowledge hiding behaviors.

H4: Psychological availability is negatively related to knowledge hiding.

### 2.3 The moderating role of person-organization fit

According to Social Identity Theory (SIT), individuals construct their self-concept through their membership in social groups and reinforce their sense of belonging by aligning with group values (Tajfel, 1974). Within organizational contexts, this theory extends to the concept of person-organization fit, which refers to the degree of alignment between individuals and organizations in terms of values, goals, and culture (Hoffman and Woehr, 2006). As artificial intelligence technologies rapidly permeate organizational environments, the influence of such fit on employees' psychological states and behaviors becomes particularly salient. When person-organization fit is high, employees' organizational identification is strengthened (Lee et al., 2015), leading them to perceive their work as a contribution to organizational objectives and mission, thereby fostering a closer psychological connection to their roles (Ashforth et al., 2008). This enhanced identification arising from high fit facilitates employees' perception of AI technology implementation as part of organizational strategy rather than a threat to their professional identity, thereby mitigating the depletion of psychological resources (Jahanzeb et al., 2021).

Employees whose values closely align with organizational culture tend to interpret AI implementation as an integral part of strategic change rather than as a threat to their individual careers. This perspective reduces psychological resistance and alleviates the depletion of psychological availability, which refers to the physiological, emotional, and cognitive resources accessible to individuals (Brown, 2000; Hogg and Terry, 2000). Furthermore, individuals with high person-organization fit are more likely to build knowledge-sharing networks with colleagues, where mutual assistance compensates for resource loss and helps maintain the resources necessary for psychological availability (Kristof, 1996; May et al., 2004). In contrast, employees with low person-organization fit may lack organizational identification and thus perceive AI technology as an obstacle to career development, resulting in excessive depletion of psychological resources and reduced psychological availability (Hobfoll, 1989). Therefore, person-organization fit moderates the relationship between employees' AI awareness and psychological availability.

H5: High person-organization fit attenuates the negative effect of AI awareness on psychological availability, whereas low person-organization fit strengthens this negative effect.

Moreover, when individuals exhibit a high degree of alignment with organizational values, they are more likely to incorporate organizational identity into their self-concept, whereby this sense of belonging shapes their behavioral norms (Tajfel, 1974). Person-organization fit essentially reflects the congruence between individuals and organizations across dimensions such as values and goals (Kristof-Brown et al., 2005).

First, increased person-organization fit enhances the consistency of event evaluations between individuals and organizations, thereby fostering a shared understanding that optimizes communication effectiveness and strengthens mutual trust (Edwards and Cable, 2009). Prior research has demonstrated that distrust among members is a critical antecedent of knowledge hiding behavior, whereas the establishment of trust significantly reduces such behavior (Wang D. Y. et al., 2024). Second, high similarity reflected in shared values increases mutual attraction between organizations and individuals (Sluss and Ashforth, 2008). This attraction further consolidates trust and facilitates communication, thereby indirectly inhibiting the tendency to hide knowledge. Finally, higher levels of person-organization fit promote organizational identification, leading individuals to perceive themselves as situated within a supportive organizational environment. Such a supportive environment not only enhances employees' resilience and hope in coping with uncertainties related to AI technology but also reduces the occurrence of counterproductive behaviors by encouraging positive actions (Avey et al., 2011; Edwards and Cable, 2009; Luthans et al., 2007; Oo et al., 2018).

Specifically, individuals with higher person-organization fit are more willing to engage in knowledge sharing to achieve common goals rather than resorting to knowledge hiding to maintain personal competitive advantages. In contrast, individuals with lower person-organization fit tend to exhibit lower levels of trust toward the organization, lack effective communication, and perceive insufficient support, which increases their likelihood of engaging in knowledge hiding behaviors to preserve their own competitive advantage. Therefore, this study proposes that person-organization fit moderates the mediating effect of psychological availability in the relationship between AI awareness and employee knowledge hiding. Specifically, as person-organization fit increases, the indirect effect of AI awareness on knowledge hiding through psychological availability weakens.

H6: When person-organization fit is high, the indirect effect of AI awareness on employee knowledge hiding behavior through psychological availability is weakened; conversely, when person-organization fit is low, this indirect effect is strengthened.

**Figure 1:**
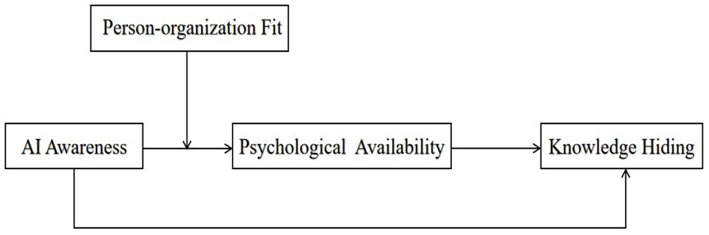
Theoretical framework.

Figure 1 illustrates the theoretical framework of the study.

## 3 Method

### 3.1 Sample and procedure

This study conducted an online survey via a web-based platform, collecting valid responses from 311 employees. Prior to the survey, the purpose, limitations, anonymity, and voluntariness of the study were clearly communicated, and each participant provided informed consent. To effectively mitigate common method bias, we followed the recommendations of Podsakoff et al. (2003) by implementing a two-wave data collection procedure with a 2-week interval between waves. At Time 1 (T1), data on participants' demographic information, AI awareness, psychological availability, person-organization fit, and control variables were collected. Two weeks later, at Time 2 (T2), the same participants were invited to complete a follow-up survey focusing on their knowledge hiding behaviors. After data screening and cleaning, 311 valid questionnaires were retained, resulting in a response rate of 77.75%. The average age of employees was 33.48 years (*SD* = 7.10), and the average tenure was 10.13 years (*SD* = 6.32). Among the respondents, 54.66% held a bachelor's degree or higher, and 46.6% were male.

### 3.2 Measures

The survey instruments in this study utilized a five-point Likert scale, with response options ranging from 1 (strongly disagree) to 5 (strongly agree). Following Brislin (1970) rigorous translation guidelines, established scales from leading international journals with demonstrated reliability and validity were adopted. All scales were translated into Chinese through a standard forward-backward translation process and subsequently reviewed by scholars and colleagues specializing in the relevant fields to finalize the measurement items. After conducting statistical analyses on the collected data, the reliability of all scales was confirmed to be satisfactory, with Cronbach's alpha coefficients exceeding 0.80.

AI awareness was measured using a four-item scale developed by Brougham and Haar (2018), with a representative item stating, “I believe my job position could be replaced by AI,” and exhibited a Cronbach's alpha of 0.93. Psychological availability was assessed with a five-item scale developed by May et al. (2004), including items such as “I believe I can adapt to competition in my work,” with a Cronbach's alpha of 0.85. Person-organization fit was measured using a five-item scale developed by Resick et al. (2007), with sample items like “My values align with those of my organization and colleagues,” yielding a Cronbach's alpha of 0.94. Employee knowledge hiding behavior was assessed using a twelve-item scale developed by Connelly et al. (2012), featuring items such as “I agree to help others but provide incorrect information,” with a Cronbach's alpha of 0.84. Additionally, gender, age, education level, tenure, position, job role, industry, and monthly income were included as control variables.

## 4 Results

This study employed *SPSS 27.0* and *AMOS 29.0* for statistical analyses. Specifically, confirmatory factor analysis (CFA) and structural equation modeling (SEM) were conducted using *AMOS 29.0*. Descriptive statistics, correlation analyses, and hierarchical regression analyses were performed with *SPSS 27.0*. Additionally, moderation and mediation effects were tested using the *PROCESS macro 4.0* with 5,000 bootstrap samples.

Table 1 reports the means, standard deviations, and intercorrelations among the study variables, with Cronbach's alpha coefficients for the four focal constructs ranging from 0.838 to 0.934 (values presented in parentheses). As shown in Table 2, the composite reliability (CR) values ranged from 0.838 to 0.934, and the average variance extracted (AVE) values ranged from 0.512 to 0.621, all meeting established psychometric standards. Harman's single-factor test showed the first factor accounted for 32.3% of the total variance, falling below the accepted 40% threshold. This indicates common method bias is unlikely to be a significant issue. Additionally, the variance inflation factor (VIF) values for all variables ranged from 1.05 to 3.12, which are substantially lower than the threshold of 10, indicating that multicollinearity is not a serious issue and does not compromise the validity of the statistical results (Podsakoff et al., 2003).

**Table 1:** Descriptive statistics and correlations among study variables.

**Variable**	***M***	***SD***	**1**	**2**	**3**	**4**	**5**	**6**	**7**	**8**	**9**	**10**	**11**	**12**
1. Sex	1.53	0.50	—											
2. Age	2.87	1.02	0.006	—										
3. Education	2.57	0.84	0.008	−0.032	—									
4. Work time	2.58	1.40	−0.003	0.780^**^	0.042	—								
5. Position level	1.67	0.82	0.003	0.195^**^	0.166^**^	0.244^**^	—							
6. Position	2.87	1.23	−0.011	0.020	0.086	0.026	0.056	—						
7. Industry	5.54	2.89	0.02	−0.023	−0.072	−0.054	−0.019.	−0.063	—					
8. Income	1.86	0.75	−0.018	0.482^**^	0.083	0.513^**^	0.455^**^	0.123^*^	−0.056	—				
9. *AA*	3.51	1.13	−0.085	−0.006	−0.016	−0.010	−0.130^*^	−0.007	−0.043	0.036	(0.867)			
10. *PA*	3.40	1.10	0.068	−0.073	−0.032	0.083	0.100	−0.057	0.067	−0.035	−0.461^**^	(0.869)		
11. *POF*	3.58	0.89	0.007	−0.074	−0.112^*^	−0.032	0.011	0.011	−0.009	−0.074	−0.209^**^	0.358^**^	(0.838)	
12. *KH*	3.60	0.97	0.031	0.037	−0.067	0.118^*^	−0.020	0.041	−0.061	0.015	0.404^**^	−0.367^**^	−0.086	(0.934)

*N* = 311; AA, AI awareness; PA, psychological availability; POF, person-organization fit; KH, knowledge hiding.

^**^p < 0.01, ^*^p < 0.05; Values in parentheses represent the internal consistency coefficients of the scales.

**Table 2:** Confirmatory factor analysis results.

**Variable**	**Factor**	**Factor loadings**	**AVE**	**CR**	**Cronbach's α**
POF	*POF1*	0.671	0.512	0.838	0.838
	*POF2*	0.765			
	*POF3*	0.703			
	*POF4*	0.702			
	*POF5*	0.725			
AA	*AA1*	0.785	0.621	0.867	0.867
	*AA2*	0.781			
	*AA3*	0.772			
	*AA4*	0.813			
PA	*PA1*	0.733	0.571	0.869	0.871
	*PA2*	0.775			
	*PA3*	0.721			
	*PA4*	0.782			
	*PA5*	0.769			
KH	*KH1*	0.750	0.540	0.934	0.934
	*KH2*	0.738			
	*KH3*	0.735			
	*KH4*	0.732			
	*KH5*	0.741			
	*KH6*	0.713			
	*KH7*	0.711			
	*KH8*	0.749			
	*KH9*	0.754			
	*KH10*	0.745			
	*KH11*	0.752			
	*KH12*	0.700			

AA, AI awareness; PA, psychological availability; POF, person-organization fit; KH, knowledge hiding.

The confirmatory factor analysis results presented in Table 3 indicate that the four-factor model consisting of AI awareness, psychological availability, person and organization fit, and knowledge hiding demonstrated the best fit (χ^2^*/df* = 1.254, *RMSEA* = 0.029, *CFI* = 0.982, *TLI* = 0.980). This model fit was significantly better than the three-factor model (χ^2^*/df* = 1.253), the two-factor model (χ^2^*/df* = 2.720), and the single-factor model (χ^2^*/df* = 5.248), thereby confirming satisfactory discriminant validity among the variables.

**Table 3:** Results of confirmatory factor analysis.

**Model**	***χ2/df***	***RMSEA***	***NFI***	***TLI***	***CFI***
Four-factor model (*AA; PA; POF; KH*)	1.254	0.029	0.917	0.980	0.982
Three-factor model (*AA+PA; POF; KH*)	2.507	0.070	0.832	0.880	0.891
Two-factor model (*AA+PA+POF; KH*)	3.974	0.098	0.731	0.763	0.783
One-factor model (*AA+PA+POF+KH*)	6.502	0.133	0.559	0.562	0.597

*N* = 311. AA, AI awareness; PA, psychological availability; POF, person-organization fit; KH, knowledge hiding.

Hierarchical regression analysis results presented in Table 4 indicate that AI awareness significantly and positively predicts knowledge hiding (Model 3: β = 0.359, *p* < 0.001), thereby supporting Hypothesis H1. AI awareness significantly and negatively influences psychological availability (Model 1: β = −0.442, *p* < 0.001), whereas psychological availability negatively predicts knowledge hiding (Model 4: β = −0.322, *p* < 0.001), thus providing support for Hypotheses H3 and H4.

**Table 4:** Hierarchical regression analysis results.

**Variable**	***PA***	***PA***	***KH***	***KH***	***KH***	***KH***
	**Model 1**	**Model 2**	**Model 3**	**Model 4**	**Model 5**	**Model 6**
1. Sex	0.063	0.090	0.132	0.113	0.144	0.146
2. Age	−0.030	0.014	−0.117	−0.132	−0.123	−0.119
3. Education	−0.047	−0.005	−0.066	−0.093	−0.075	−0.069
4. Work time	−0.070	−0.098	0.178^**^	0.151^**^	0.164^**^	0.160^**^
5. Position level	0.076	0.085	0.030	−0.002	0.045	0.046
6. Position	−0.057	−0.057	0.034	0.012	0.023	0.022
7. Industry	0.014	0.015	−0.013	−0.012	−0.011	−0.010
8. Income	0.032	0.045	−0.121	−0.066	−0.115	−0.110
9. *AA*	−0.442^***^	−0.387^***^	0.359^***^		0.272^***^	
10. *PA*				−0.322^***^	−0.197^***^	−0.211^***^
11. *POF*		0.277^***^				0.054
12. *PA × POF*		0.158^**^				
13. *R^2^*	0.232	0.322	0.201	0.240	0.322	0.242
14. *ΔR^2^*	0.197	0.090	0.166	0.038	0.019	0.002
15. *F*	10.099	12.907	8.437	9.449	12.907	8.659
16. *ΔF*	77.007	19.852	62.477	15.021	8.527	0.820

^***^p < 0.001, ^**^p < 0.01.

When AI awareness and psychological availability were simultaneously included in the model (Model 5), the direct effect of AI awareness on knowledge hiding remained significant but decreased by 24.2% (β = 0.359–0.272), while psychological availability maintained a significant negative effect (β = −0.197, *p* < 0.001). Furthermore, the Bootstrap analysis (Table 5) confirmed that the indirect effect of psychological availability was 0.0917 [*SE* = 0.0255, 95% *CI* = (0.0459, 0.1448)], and the direct effect was also significant [*effect* = 0.2570, *SE* = 0.0495, 95% *CI* = [0.1596, 0.3543)], indicating that psychological availability partially mediates the relationship between AI awareness and knowledge hiding, thereby supporting Hypothesis H2.

**Table 5:** Bootstrap analysis of mediation effects.

**Effect**	**Effect value**	**Boot SE**	**LLCI**	**ULCI**
Indirect effect	0.0917	0.0255	0.0459	0.1448
Direct effect	0.2570	0.0495	0.1596	0.3543

**Figure 2:**
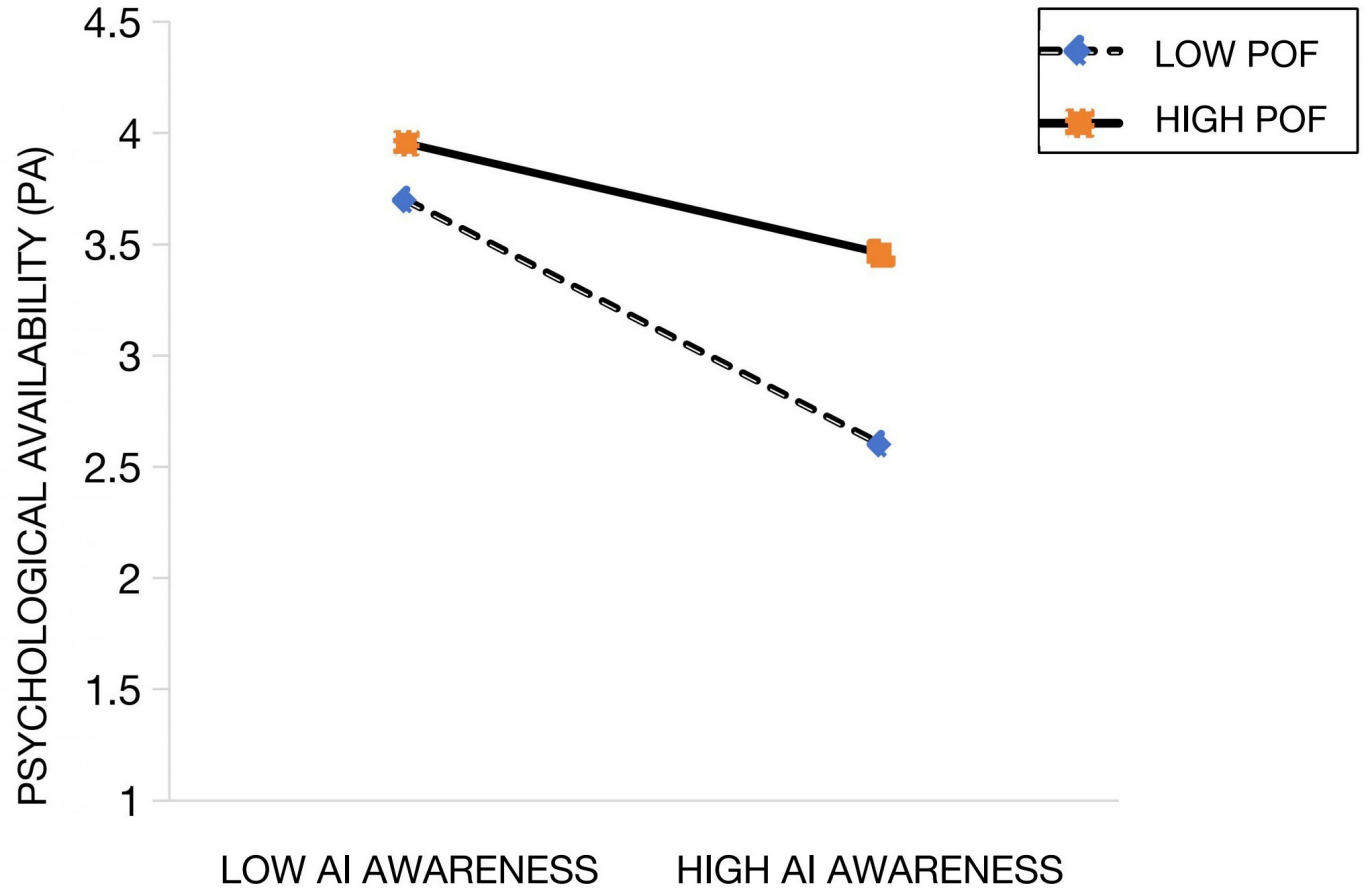
The moderating role of person-organization fit.

To test Hypothesis H5, the independent variable and the moderator were first mean-centered. The interaction term was then created by multiplying the mean-centered independent variable with the moderator, followed by hierarchical regression analysis. As shown in Model 2 of Table 4, after controlling for the main effects of AI awareness and person-organization fit, the interaction term significantly predicted employees' knowledge hiding behavior (*b* = 0.158, *p* < 0.01, Δ*R*^2^ = 0.090), indicating a significant moderating effect of person-organization fit on the relationship between AI awareness and psychological availability. Furthermore, simple slope analyses were conducted by grouping person-organization fit into high and low levels, defined as one standard deviation above and below the mean, respectively. As illustrated in Figure 2, when person-organization fit was low, AI awareness significantly and negatively predicted psychological availability (β = −0.532, *p* < 0.001). When person-organization fit was high, the negative effect of AI awareness on psychological availability remained significant but was attenuated (β = −0.264, *p* < 0.001). These empirical results suggest that a higher level of person-organization fit weakens the negative relationship between AI awareness and psychological availability, thereby supporting Hypothesis H5.

Building on PROCESS 4.0 macro, hypothesis H6 was tested, with results presented in Table 6. It was found that the index of the moderated mediation effect was −0.031, with a standard error of 0.013 and a 95% confidence interval (CI) of [−0.060, −0.010], which did not include zero. This indicates that person-organization fit moderates the relationship among AI awareness, psychological availability, and employee knowledge hiding. When person-organization fit was low, the indirect effect of psychological availability on knowledge hiding was 0.108 (*SE* = 0.031), with a 95% CI of [0.053, 0.175], excluding zero. In contrast, when person-organization fit was high, the indirect effect was 0.054 (*SE* = 0.020), with a 95% CI of [0.019, 0.098], also excluding zero. Moreover, the difference in the mediation effect of psychological availability between the high and low levels of person-organization fit was significant, with an estimated difference of −0.055 (*SE* = 0.020) and a 95% CI of [−0.106, −0.019], which did not include zero. Collectively, these findings support hypothesis H6.

**Table 6:** Moderated mediation effect.

**Independent**	**Independent**			**Index of moderated mediation**	
	**Moderator**	**Effect**	**(CI)**	**Index**	**(CI)**
KH	Low POF (−1SD)	0.108	[0.053, 0.175]	−0.031	[−0.060, −0.010]
	High POF (+1SD)	0.054	[0.019, 0.098]		
	Difference (high-low)	−0.055	[−0.106, −0.019]		

## 5 Discussion

Building on the Conservation of Resources theory (Hobfoll, 1989) and the Social Identity theory (Tajfel, 1979), this study develops a theoretical framework that explains how artificial intelligence awareness influences employees' knowledge hiding behavior. Through empirical testing, the study further uncovers the underlying mechanisms and boundary conditions of this relationship. The results reveal that artificial intelligence awareness significantly exacerbates employees' tendency to engage in knowledge hiding (β = 0.359, *p* < 0.001). This finding lends empirical support to the notion that technological turbulence reinforces defensive knowledge protection (Arias-Perez and Velez-Jaramillo, 2022), while also extending prior research that has predominantly focused on individual dispositions (e.g., self-interest), knowledge characteristics (e.g., the inherent difficulty of sharing tacit knowledge), or leadership styles (e.g., abusive supervision) as antecedents of knowledge hiding (Connelly et al., 2012; Siachou et al., 2021; Shen et al., 2025).

As the core pathway of the theoretical framework, artificial intelligence awareness was found to significantly reduce employees' psychological availability for knowledge sharing by depleting their physical, emotional, and cognitive resources (β = −0.442, *p* < 0.001), which in turn prompted the adoption of knowledge hiding strategies such as playing dumb, evasive hiding, and rationalized hiding (β = −0.322, *p* < 0.001; Kahn, 1990; Halbesleben et al., 2014). This pathway differs from traditional explanations that emphasize individual motivation or organizational pressure (Restubog et al., 2011), as it elucidates the conversion of technology-induced anxiety into counterproductive behavior from the perspective of dynamic resource availability (Qian et al., 2020). By introducing this mechanism, the present study extends the application of the Conservation of Resources theory to the context of artificial intelligence, demonstrating how perceived technological threats can independently trigger defensive behaviors through psychological mechanisms rooted in resource depletion—an approach that contrasts markedly with prior research that has primarily focused on motivational or structural stressors (Restubog et al., 2011; Serenko and Bontis, 2016).

In exploring boundary conditions, it was confirmed that person-organization fit significantly moderates the relationship between artificial intelligence awareness and psychological availability (Ashforth and Mael, 1989; Edwards and Cable, 2009). Specifically, when employees' personal values were highly congruent with organizational values, the negative effect of artificial intelligence awareness on psychological availability was substantially attenuated (β = −0.264 for the high-fit group vs. β = −0.532 for the low-fit group), and the mediating role of psychological availability was correspondingly weakened, with the indirect effect decreasing from 0.108 to 0.054. These findings strongly support the propositions of Social Identity theory, wherein employees with high value congruence are more likely to cognitively reframe technological implementation as an organizational strategic initiative rather than an existential threat to the individual, thereby achieving cognitive reappraisal through internalized organizational identification (Ashforth and Mael, 1989; Edwards and Cable, 2009). Compared with prior studies focusing on moderating effects of leadership behaviors or formal organizational systems (Guan, 2016; Wang et al., 2021), this study demonstrates that value congruence fundamentally reshapes the cognitive appraisal of technological threats, providing novel theoretical insights into organizational behavior in human–AI collaboration contexts (Xiao et al., 2018; Chen et al., 2022).

### 5.1 Theoretical implications

This study demonstrates how artificial intelligence awareness, as a critical antecedent, activates knowledge hiding as a form of counterproductive behavior by depleting individuals' psychological resources (Halbesleben et al., 2014). Through this mechanism, a more refined understanding has been developed regarding employee counterproductive responses within the context of technology-driven organizational transformation (Wang et al., 2014).

In addition, this study provides the first rigorous empirical evidence supporting the mediating role of psychological availability in the relationship between artificial intelligence awareness and knowledge hiding. This finding not only affirms the central proposition of the Conservation of Resources theory, which posits that individuals tend to adopt protective strategies when perceiving resource threats (Hobfoll, 2001), but also advances the understanding of how artificial intelligence technologies influence employee behavior through underlying psychological mechanisms. By doing so, the study addresses a critical gap in the human–machine interaction literature, where empirical support for such mediating pathways has remained limited (Podsakoff et al., 2012).

Furthermore, this study extends the boundary conditions of knowledge hiding by identifying the moderating effect of person-organization fit (Serenko and Bontis, 2016), while also advancing the application of Social Identity theory into the emerging context of human–AI collaboration. Specifically, when employees perceive a high level of value congruence with their organization, the indirect effect of artificial intelligence awareness on knowledge hiding through psychological availability is significantly attenuated. This moderating effect highlights the critical buffering role of organizational context in mitigating the adverse consequences of technological transformation (Edwards and Cable, 2009). It also provides empirical support for the applicability of Social Identity theory in human-technology interaction settings, wherein employees' identification with the organization tends to reduce perceived identity and value threats triggered by artificial intelligence deployment (Blader et al., 2017). This theoretical extension not only offers a novel boundary framework for knowledge management in the age of artificial intelligence, but also promotes the evolution of Social Identity theory within digitalized work environments, thereby deepening scholarly insight into the dynamic mechanisms of human–AI collaboration.

In conclusion, this study contributes to the literature by systematically articulating the underlying mechanisms through which artificial intelligence awareness influences knowledge hiding, integrating theoretical insights with empirical validation. Specifically, the mediating role of psychological availability and the moderating role of person-organization fit jointly constitute a comprehensive theoretical framework. This framework not only advances the understanding of counterproductive work behavior in the context of technological transformation (Chi, 2009), but also offers a verifiable pathway for future research. Furthermore, it underscores the critical importance of organizational interventions that are designed to enhance the effectiveness of human–AI collaboration. Ultimately, through a logically coherent chain of reasoning, this study achieves both a theoretical extension and empirical refinement of the Conservation of Resources theory and the Social Identity theory within the context of artificial intelligence, thereby laying a solid foundation for future contributions in this emerging research domain.

### 5.2 Practical implications

Artificial intelligence awareness, characterized as an anxiety-laden cognitive response to technological change, is prevalent among employees within organizations; however, it can be alleviated through systematic interventions (Chen et al., 2022; Zhu et al., 2023). This study focuses on the underlying mechanism linking artificial intelligence awareness and employee knowledge hiding behavior, aiming to elucidate the intrinsic logic of their relationship. It intends to guide both organizations and individuals to adopt a rational stance toward technological innovation, thereby transforming artificial intelligence from a catalyst of workplace anxiety into an enabler of knowledge creation. The findings suggest that organizations can cultivate a human–AI symbiotic ecosystem by means of cognitive guidance, institutional design, and cultural shaping, which facilitates the deep integration of technology and human capital (Chen et al., 2022; Zhu et al., 2023).

From a practical intervention perspective, managers should first promote cognitive reframing among employees by repositioning artificial intelligence technology as a collaborative tool for knowledge creation rather than a threat to one's career. Systematic training programs, such as the development of human–AI collaborative knowledge co-creation platforms, can be implemented to integrate AI technology into employees' capability development systems. This approach serves to reconstruct employees' perceptions of technological change, thereby reducing defensive motivations related to resource loss and subsequently inhibiting knowledge hiding behaviors (Mo et al., 2024). This intervention logic is grounded in the Conservation of Resources theory, which posits that when employees perceive a diminished threat of artificial intelligence technology to their occupational resources, their defensive knowledge protection behaviors tend to decrease accordingly (Hobfoll, 1989).

Second, organizations should develop a culture oriented toward knowledge co-creation by enhancing the transparency and reciprocity of knowledge flows through institutional design and cultural shaping. For example, integrating knowledge sharing into performance appraisal systems can serve as an institutional incentive to promote collaborative knowledge creation among members (Connelly et al., 2019). The theoretical foundation of this practice lies in the premise that institutional incentives alter employees' cost-benefit evaluations of their behaviors, thereby weakening the intrinsic motivation to hide knowledge (Serenko and Bontis, 2016). Meanwhile, cultural shaping fosters a climate of sharing, which reduces employees' dependence on knowledge exclusivity and further facilitates knowledge exchange.

Finally, managers should strengthen employees' identification with organizational values through cultural development and employee care initiatives, thereby enhancing person-organization fit. A high level of fit tends to alleviate career uncertainty anxiety induced by technological change and reduce counterproductive behaviors such as knowledge hiding (Kristof-Brown et al., 2005). Grounded in Social Identity theory, when employees internalize organizational values as part of their self-concept, they are more likely to regulate their behavior from the perspective of organizational interests, thereby maintaining a propensity for knowledge sharing despite the impact of artificial intelligence technologies (Tajfel, 1979).

### 5.3. Limitations and future directions

First, although the scales used in this study underwent a rigorous back-translation procedure to ensure semantic accuracy, directly applying instruments developed in Western contexts to the Chinese setting may not fully capture the nuances of local management research. Future studies are encouraged to develop more culturally adapted measurement scales that incorporate China's unique culture, values, and environment to improve the reliability and validity of these tools within Chinese organizational contexts.

Second, despite employing a two-stage data collection method to mitigate common method bias, the cross-sectional nature of the data limits the examination of the dynamic effects of artificial intelligence awareness on knowledge hiding behavior over time. Longitudinal research using multi-wave data collection would allow observation of changes in employees' knowledge hiding behavior at different phases of AI implementation, such as the initial deployment, adaptation transition, and mature application stages, thereby offering a more nuanced understanding of the underlying mechanisms.

Finally, this study focused solely on the moderating role of person-organization fit and did not consider other potential moderating variables. Future research could explore additional contextual moderators to further delineate the boundary conditions under which artificial intelligence awareness influences employee knowledge hiding behavior.

## 6 Conclusion

This study is grounded in the practical context of artificial intelligence technology's deep infiltration into organizational settings. By integrating the Conservation of Resources theory and Social Identity theory, it systematically reveals the underlying mechanisms and boundary conditions through which artificial intelligence awareness influences employee knowledge hiding behavior. This offers novel theoretical insights and practical avenues for understanding the knowledge management challenges in the era of human–machine collaboration. The findings indicate that employees' perceived threat of job displacement by artificial intelligence technology leads to the depletion of their physiological, emotional, and cognitive resources, thereby reducing psychological availability and triggering defensive knowledge hiding behaviors such as evasive and feigning ignorance tactics (Connelly et al., 2012; Hobfoll, 1989). This transmission pathway suggests that in emerging work environments characterized by algorithm-human collaboration, the perception of technological threat reshapes employees' willingness to share knowledge via resource depletion mechanisms, thereby exerting reconstructive pressure on traditional organizational knowledge management paradigms.

From a theoretical standpoint, this study is the first to empirically examine the mediating role of psychological availability in the relationship between artificial intelligence technology and knowledge hiding. It extends Kahn (1990) concept of psychological availability to the context of technological change, confirming that individuals' subjective perception of their resource availability serves as a critical bridge linking AI threat awareness and counterproductive behaviors. Additionally, the findings demonstrate that person-organization fit, by strengthening employees' organizational identification, attenuates the negative impact of AI awareness on psychological availability. This conclusion not only validates the applicability of Social Identity theory (Tajfel, 1979) within human–machine collaboration contexts but also reveals the unique role of value congruence as an organizational contextual factor in buffering technological shocks and maintaining knowledge flow (Kristof, 1996). These insights surpass the traditional knowledge hiding literature that predominantly focuses on individual traits or leadership styles, thereby providing important theoretical support for knowledge management innovation in the AI era.

From the perspective of practical implications, the research findings offer direct guidance for organizations addressing the knowledge-sharing challenges arising from artificial intelligence (AI) technology. Building on the mechanism whereby AI awareness influences knowledge hiding through psychological availability, managers are advised to develop human and machine collaboration training programs to help employees reconstruct their perceptions of AI technology, whereby AI is redefined from a career threat to a knowledge co-creation partner, thereby reducing defensive knowledge protection motivation (Mo et al., 2024). Simultaneously, organizations should focus on cultivating a culture characterized by high person to organization fit, wherein value transmission and institutional design, such as incorporating knowledge sharing into performance appraisal, strengthen employees' organizational identification, enabling them to maintain a behavioral logic that prioritizes organizational welfare amid technological change (Ashforth and Mael, 1989). This dual-faceted intervention strategy not only aligns with the Conservation of Resources theory's explanation of individuals' motivation to protect resources but also operationalizes the Social Identity theory's requirements for constructing organizational identification, thereby providing a viable pathway for fostering a knowledge-sharing ecosystem characterized by human and machine collaboration.

The study further highlights three areas for future research. First, it is essential to develop measurement instruments with greater cultural adaptability, since existing Western scales may not fully capture indigenous dimensions, such as those in Chinese organizations, which influence knowledge hiding (Huang and Gursoy, 2024). Second, longitudinal studies are needed to track the dynamic changes in employees' knowledge hiding behaviors across different stages of AI technology implementation, thereby revealing more detailed patterns of behavioral evolution (Kim and Kim, 2024). Third, boundary conditions can be extended by investigating the moderating roles of factors such as algorithms and leadership in the relationship between technology cognition and knowledge hiding (Mahmud et al., 2022; Wang H. et al., 2024). Knowledge management in the era of artificial intelligence fundamentally involves organizational cognitive reconstruction, whereby only through deeply integrating technological values into employees' belief systems can AI awareness transform from a trigger of knowledge hiding into a catalyst of knowledge co-creation. This represents not only an important direction for future research but also a critical process through which organizations can achieve human and machine symbiosis amid digital transformation.

## Data Availability

The raw data supporting the conclusions of this article will be made available by the authors, without undue reservation.

## References

[B1] Abiemo M. K. Azila-Gbettor E. M. Atatsi E. A. Honyenuga B. Q. Mensah C. (2024). *Predicting project performance from occupational stress, psychological availability and ethical* leadership: moderated-mediation and mediated-moderation models. Eng. Constr. Archit. Manag. 32, 1–23. 10.1108/ECAM-04-2024-0502

[B2] Amabile T. M. Barsade S. G. Mueller J. S. Staw B. M. (2005). Affect and creativity at work. Adm. Sci. Quart. 50, 367–403. 10.2189/asqu.2005.50.3.36721821037

[B3] Arias-Pérez J. Huynh T. (2023). Flipping the odds of AI-driven open innovation: the effectiveness of partner trustworthiness in counteracting interorganizational knowledge hiding. Indus. Mark. Manag. 111, 30–40. 10.1016/j.indmarman.2023.03.005

[B4] Arias-Perez J. Velez-Jaramillo J. (2022). Understanding knowledge hiding under technological turbulence caused by artificial intelligence and robotics. J. Knowl. Manag. 26, 1476–1491. 10.1108/JKM-01-2021-0058

[B5] Ashforth B. E. Harrison S. H. Corley K. G. (2008). Identification in organizations: an examination of four fundamental questions. J. Manage. 34, 325–374. 10.1177/0149206308316059

[B6] Ashforth B. E. Mael F. (1989). Social identity theory and the organization. Acad. Manag. Rev. 14, 20–39. 10.2307/258189

[B7] Avey J. B. Avolio B. J. Crossley C. D. Luthans F. (2009). Psychological ownership: theoretical extensions, measurement and relation to work outcomes. J. Organ. Behav. 30, 173–191. 10.1002/job.583

[B8] Avey J. B. Luthans F. Youssef C. M. (2010). The additive value of positive psychological capital in predicting work attitudes and behaviors. J. Manage. 36, 430–452. 10.1177/0149206308329961

[B9] Avey J. B. Reichard R. J. Luthans F. Mhatre K. H. (2011). Meta-analysis of the impact of positive psychological capital on employee attitudes, behaviors, and performance. Hum. Resour. Dev. Quart. 22, 127–152. 10.1002/hrdq.20070

[B10] Avey J. B. Wernsing T. S. Luthans F. (2008). Can positive employees help positive organizational change? Impact of psychological capital and emotions on relevant attitudes and behaviors. J. Appl. Behav. Sci. 44, 48–70. 10.1177/0021886307311470

[B11] Binyamin G. Carmeli A. (2010). Does structuring of human resource management processes enhance employee creativity? The mediating role of psychological availability. *Hum. Resour. Manage*. 49, 999–1024. 10.1002/hrm.20397

[B12] Bjorntorp P. (2001). Do stress reactions cause abdominal obesity and comorbidities? Obes. Rev. 2, 73–86. 10.1046/j.1467-789x.2001.00027.x 12119665

[B13] Blader S. L. Patil S. Packer D. J. (2017). Organizational identification and workplace behavior: more than meets the eye. Res. Organ. Behav. 37, 19–34. 10.1016/j.riob.2017.09.001

[B14] Brickson S. L. (2013). The impact of organizational identity orientation on individual-level organizational citizenship behavior. Organ. Sci. 24, 870–888. 10.1287/orsc.1110.073035126227

[B15] Brislin R. W. (1970). Back-translation for cross-cultural research. *J. Cross Cult. Psychol*. 1, 185–216.

[B16] Brougham D. Haar J. (2018). Smart technology, artificial intelligence, robotics, and algorithms (STARA): employees' perceptions of our future workplace. J. Manag. Organ. 24, 239–257. 10.1017/jmo.2016.55

[B17] Brown R. (2000). Social identity theory: past achievements, current problems and future challenges. Eur. J. Soc. Psychol. 30, 745–778. 10.1002/1099-0992(200011/12)30:6<745::AID-EJSP24>3.0.CO;2-O

[B18] Byrne Z. Albert L. Manning S. Desir R. (2017). Relational models and engagement: an attachment theory perspective. J. Manag. Psychol. 32, 30–44. 10.1108/JMP-01-2016-0006

[B19] Cable D. M. Edwards J. R. (2004). Complementary and supplementary fit: a theoretical and empirical integration. J. Appl. Psychol. 89, 822–834. 10.1037/0021-9010.89.5.822 15506863

[B20] Cabrera A. Collins W. C. Salgado J. F. (2006). Determinants of individual engagement in knowledge sharing. Int. J. Hum. Resour. Manag. 17, 245–264. 10.1080/09585190500404614

[B21] Cai Z. Huang Q. Liu H. Wang X. (2018). Improving the agility of employees through enterprise social media: The mediating role of psychological conditions. *Int. J. Inf. Manage*. 38, 52–63. 10.1016/j.ijinfomgt.2017.09.001

[B22] Carlson D. S. Frone M. R. (2003). Relation of behavioral and psychological involvement to a new four-factor conceptualization of work-family interference. J. Bus. Psychol. 17, 515–535. 10.1023/A:1023404302295

[B23] Cerne M. Nerstad C. G. L. Dysvik A. Skerlavaj M. (2014). What goes around comes around: knowledge hiding, perceived motivational climate, and creativity. Acad. Manag. J. 57, 172–192. 10.5465/amj.2012.0122

[B24] Chen C. Zhu L. Liu C. Xu S. (2022). The theory of symbiosis: a new management paradigm in the digital age. Foreign Econ. Manag. 44, 68–83. 10.16538/j.cnki.fem.20211206.101

[B25] Chi M. T. H. (2009). Active-constructive-interactive: a conceptual framework for differentiating learning activities. Top. Cogn. Sci. 1, 73–105. 10.1111/j.1756-8765.2008.01005.x 25164801

[B26] Connelly C. E. Cerne M. Dysvik A. Skerlavaj M. (2019). Understanding knowledge hiding in organizations. J. Organ. Behav. 40, 779–782. 10.1002/job.2407

[B27] Connelly C. E. Zweig D. (2015). How perpetrators and targets construe knowledge hiding in organizations. Eur. J. Work Organ. Psychol. 24, 479–489. 10.1080/1359432X.2014.931325

[B28] Connelly C. E. Zweig D. Webster J. Trougakos J. P. (2012). Knowledge hiding in organizations. J. Organ. Behav. 33, 64–88. 10.1002/job.737

[B29] Danner-Vlaardingerbroek G. Kluwer E. S. van Steenbergen E. F. van der Lippe T. (2013). Knock, knock, anybody home? Psychological availability as link between work and relationship. Pers. Relatsh. 20, 52–68. 10.1111/j.1475-6811.2012.01396.x

[B30] Edwards J. R. Cable D. A. (2009). The value of value congruence. J. Appl. Psychol. 94, 654–677. 10.1037/a0014891 19450005

[B31] Erdogan B. Bauer T. N. (2009). Perceived overqualification and its outcomes: the moderating role of empowerment. J. Appl. Psychol. 94, 557–565. 10.1037/a0013528 19271809

[B32] Erdogan B. Karaeminogullari A. T. Bauer T. N. Ellis A. M. (2020). Perceived overqualification at work: implications for extra-role behaviors and advice network centrality. J. Manage. 46, 583–606. 10.1177/0149206318804331

[B33] Glikson E. Woolley A. W. (2020). Human trust in artificial intelligence: review of empirical research. Acad. Manag. Ann. 14, 627–660. 10.5465/annals.2018.0057

[B34] Guan C. (2016). Research on inclusive leadership from the perspective of moral capital. J. Capl. Norm. Univ. 4, 66–71.

[B35] Haefner N. Wincent J. Parida V. Gassmann O. (2021). Artificial intelligence and innovation management: a review, framework, and research agenda. Technol. Forecast. Soc. Transform. 162:120392. 10.1016/j.techfore.2020.120392

[B36] Halbesleben J. R. B. Neveu J.-P. Paustian-Underdahl S. C. Westman M. (2014). Getting to the “COR”: understanding the role of resources in conservation of resources theory. J. Manage. 40, 1334–1364. 10.1177/0149206314527130

[B37] He P. Jiang C. Xu Z. Shen C. (2021). Knowledge hiding: current research status and future research directions. Front. Psychol. 12:748237. 10.3389/fpsyg.2021.748237 34777143 PMC8586422

[B38] He Q. Liu M. Li X. (2024). Will artificial intelligence trigger employees' knowledge hiding behavior? from the theoretical perspective of relative deprivation. Foreign Econ. Manag. 46, 55–70. 10.16538/j.cnki.fem.20231226.101

[B39] Hernaus T. Cerne M. Connelly C. Vokic N. P. Skerlavaj M. (2019). Evasive knowledge hiding in academia: when competitive individuals are asked to collaborate. J. Knowl. Manag. 23, 597–618. 10.1108/JKM-11-2017-0531

[B40] Hobfoll S. E. (1989). Conservation of resources. A new attempt at conceptualizing stress. Am. Psychol. 44, 513–524. 10.1037/0003-066X.44.3.513 2648906

[B41] Hobfoll S. E. (2001). The influence of culture, community, and the nested-self in the stress process: advancing conservation of resources theory. Appl. Psychol. 50, 337–421. 10.1111/1464-0597.00062

[B42] Hobfoll S. E. Halbesleben J. Neveu J.-P. Westman M. (2018). Conservation of resources in the organizational context: the reality of resources and their consequences. Ann. Rev. Organ. Psychol. Organ. Behav. 5, 103–128. 10.1146/annurev-orgpsych-032117-104640

[B43] Hoffman B. J. Woehr D. J. (2006). A quantitative review of the relationship between person-organization fit and behavioral outcomes. J. Vocat. Behav. 68, 389–399. 10.1016/j.jvb.2005.08.003

[B44] Hogg M. A. Terry D. J. (2000). Social identity and self-categorization processes in organizational contexts. Acad. Manag. Rev. 25, 121–140. 10.2307/259266

[B45] Huang Y. Gursoy D. (2024). How does AI technology integration affect employees' proactive service behaviors? A transactional theory of stress perspective. J. Retail. Consum. Serv. 77:103700. 10.1016/j.jretconser.2023.103700

[B46] Huo W. Cai Z. Luo J. Men C. Jia R. (2016). Antecedents and intervention mechanisms: a multi-level study of RandD team's knowledge hiding behavior. J. Knowl. Manag. 20, 880–897. 10.1108/JKM-11-2015-0451

[B47] Jahanzeb S. De Clercq D. Fatima T. (2021). Organizational injustice and knowledge hiding: the roles of organizational dis-identification and benevolence. Manag. Decis. 59, 446–462. 10.1108/MD-05-2019-0581

[B48] Jha J. K. Varkkey B. (2018). Are you a cistern or a channel? Exploring factors triggering knowledge hiding behavior at the workplace: evidence from the Indian RandD professionals. J. Knowl. Manag. 22, 824–849. 10.1108/JKM-02-2017-0048

[B49] Kahn W. A. (1990). Psychological conditions of personal engagement and disengagement at work. Acad. Manag. J. 33, 692–724. 10.2307/256287

[B50] Karahanna E. Straub D. W. (1999). The psychological origins of perceived usefulness and ease-of-use. Inf. Manag. 35, 237–250. 10.1016/S0378-7206(98)00096-2

[B51] Kim B.-J. Kim M.-J. (2024). How artificial intelligence-induced job insecurity shapes knowledge dynamics: the mitigating role of artificial intelligence self-efficacy. J. Innov. Knowl. 9:100590. 10.1016/j.jik.2024.100590

[B52] Kristof A. L. (1996). Person-organization fit: an integrative review of its conceptualizations, measurement, and implications. Pers. Psychol. 49, 1–49. 10.1111/j.1744-6570.1996.tb01790.x

[B53] Kristof-Brown A. L. Zimmerman R. D. Johnson E. C. (2005). Consequences of individuals' fit at work: a meta-analysis of person-job, person-organization, person-group, and person-supervisor fit. Pers. Psychol. 58, 281–342. 10.1111/j.1744-6570.2005.00672.x

[B54] Lee E.-S. Park T.-Y. Koo B. (2015). Identifying organizational identification as a basis for attitudes and behaviors: a meta-analytic review. Psychol. Bull. 141, 1049–1080. 10.1037/bul0000012 25984729

[B55] Lee R. T. Ashforth B. E. (1996). A meta-analytic examination of the correlates of the three dimensions of job burnout. J. Appl. Psychol. 81, 123–133. 10.1037/0021-9010.81.2.123 8603909

[B56] Li J. Bonn M. A. Ye B. H. (2019). Hotel employee's artificial intelligence and robotics awareness and its impact on turnover intention: the moderating roles of perceived organizational support and competitive psychological climate. Tour. Manag. 73, 172–181. 10.1016/j.tourman.2019.02.006

[B57] Li Y. Tan C. H. (2013). Matching business strategy and CIO characteristics: the impact on organizational performance. J. Bus. Res. 66, 248–259. 10.1016/j.jbusres.2012.07.017

[B58] Lingmont D. N. J. Alexiou A. (2020). The contingent effect of job automating technology awareness on perceived job insecurity: exploring the moderating role of organizational culture. Technol. Forecast. Soc. Transform. 161:120302. 10.1016/j.techfore.2020.120302

[B59] Liu P. Yuan L. Jiang Z. (2025). The dark side of algorithmic management: investigating how and when algorithmic management relates to employee knowledge hiding? J. Knowl. Manag. 29, 342–371. 10.1108/JKM-04-2024-0507

[B60] Luthans F. Avolio B. J. Avey J. B. Norman S. M. (2007). Positive psychological capital: measurement and relationship with performance and satisfaction. Pers. Psychol. 60, 541–572. 10.1111/j.1744-6570.2007.00083.x

[B61] Mahmud H. Islam A. K. M. N. Ahmed S. I. Smolander K. (2022). What influences algorithmic decision-making? A systematic literature review on algorithm aversion. Technol. Forecast. Soc. Transform. 175:121390. 10.1016/j.techfore.2021.121390

[B62] May D. R. Gilson R. L. Harter L. M. (2004). The psychological conditions of meaningfulness, safety and availability and the engagement of the human spirit at work. J. Occup. Organ. Psychol. 77, 11–37. 10.1348/096317904322915892

[B63] Mo Z. Liu M. T. Ma Y. (2024). How AI awareness can prompt service performance adaptivity and technologically-environmental mastery. Tour. Manag. 105:104971. 10.1016/j.tourman.2024.104971

[B64] Oo E. Y. Jung H. Park I.-J. (2018). Psychological factors linking perceived CSR to OCB: the role of organizational pride, collectivism, and person-organization fit. Sustainability 10:2481. 10.3390/su10072481

[B65] Podsakoff P. M. MacKenzie S. B. Lee J.-Y. Podsakoff N. P. (2003). Common method biases in behavioral research: a critical review of the literature and recommended remedies. J. Appl. Psychol. 88, 879–903. 10.1037/0021-9010.88.5.879 14516251

[B66] Podsakoff P. M. MacKenzie S. B. Podsakoff N. P. (2012). Sources of method bias in social science research and recommendations on how to control it. Annu. Rev. Psychol. 63, 539–569. 10.1146/annurev-psych-120710-100452 21838546

[B67] Qian S. Yuan Q. Lim V. K. G. Niu W. Liu Z. (2020). Do job-insecure leaders perform less transformational leadership? The roles of emotional exhaustion and trait mindfulness. J. Lead. Organ. Stud. 27, 376–388. 10.1177/1548051820938327

[B68] Rampersad G. (2020). Robot will take your job: innovation for an era of artificial intelligence. J. Bus. Res. 116, 68–74. 10.1016/j.jbusres.2020.05.019

[B69] Resick C. J. Baltes B. B. Shantz C. W. (2007). Person-organization fit and work-related attitudes and decisions: examining interactive effects with job fit and conscientiousness. J. Appl. Psychol. 92, 1446–1455. 10.1037/0021-9010.92.5.1446 17845097

[B70] Restubog S. L. D. Scott K. L. Zagenczyk T. J. (2011). When distress hits home: the role of contextual factors and psychological distress in predicting employees' responses to abusive supervision. J. Appl. Psychol. 96, 713–729. 10.1037/a0021593 21280933

[B71] Russell G. Lightman S. (2019). The human stress response. Nat. Rev. Endocrinol. 15, 525–534. 10.1038/s41574-019-0228-0 31249398

[B72] Santhose S. S. Lawrence L. N. (2023). Understanding the implementations and limitations in knowledge management and knowledge sharing using a systematic literature review. Curr. Psychol. 42, 32427–32442. 10.1007/s12144-022-04115-6

[B73] Serenko A. Bontis N. (2016). Understanding counterproductive knowledge behavior: Antecedents and consequences of intra-organizational knowledge hiding. Journal of Knowledge Management 20, 1199–1224. 10.1108/JKM-05-2016-0203

[B74] Shalley C. E. Gilson L. L. Blum T. C. (2000). Matching creativity requirements and the work environment: Effects on satisfaction and intentions to leave. *Acad. Manage. J*. 43, 215–223. 10.2307/1556378

[B75] Shalley C. E. Zhou J. Oldham G. R. (2004). The effects of personal and contextual characteristics on creativity: where should we go from here? J. Manage. 30, 933–958. 10.1016/j.jm.2004.06.007

[B76] Shen Y. Lythreatis S. Singh S. K. Cooke F. L. (2025). A meta-analysis of knowledge hiding behavior in organizations: antecedents, consequences, and boundary conditions. J. Bus. Res. 186:114963. 10.1016/j.jbusres.2024.114963

[B77] Siachou E. Trichina E. Papasolomou I. Sakka G. (2021). Why do employees hide their knowledge and what are the consequences? A systematic literature review. J. Bus. Res. 135, 195–213. 10.1016/j.jbusres.2021.06.031

[B78] Sluss D. M. Ashforth B. E. (2008). How relational and organizational identification converge: processes and conditions. Organ. Sci. 19, 807–823. 10.1287/orsc.1070.034919642375

[B79] Sun Y. Ayub A. Fatima T. Aslam H. D. Bahoo S. (2022). The knowledge hiding loop: exploring the boundary conditions. Kybernetes 51, 3320–3339. 10.1108/K-04-2021-0307

[B80] Tajfel H. (1974). Social identity and intergroup behaviour. Soc. Sci. Inform. 13, 65–93. 10.1177/053901847401300204

[B81] Tajfel H. (1979). Individuals and groups in social psychology. Br. J. Soc. Clin. Psychol. 18, 183–190. 10.1111/j.2044-8260.1979.tb00324.x

[B82] Vrontis D. Christofi M. Pereira V. Tarba S. Makrides A. Trichina E. (2022). Artificial intelligence, robotics, advanced technologies and human resource management: a systematic review. Int. J. Hum. Resour. Manag. 33, 1237–1266. 10.1080/09585192.2020.1871398

[B83] Wang C. Wei Y. Zhao X. Zhang X. Peng Y. (2021). Abusive supervision and creativity: investigating the moderating role of performance improvement attribution and the mediating role of psychological availability. Front. Psychol. 12:658743. 10.3389/fpsyg.2021.658743 34234710 PMC8255386

[B84] Wang D. Y. Zhao J. Cui Y. M. Gao X. C. (2024). How does coworker knowledge hiding affect employee innovative behavior? Striving for progress or retreating in defeat. Sci. Technol. Prog. Policy 41, 130–140.

[B85] Wang H. Yu J. (2022). Research on the influence mechanism of sense of power on deviant innovation: an interpretation based on chinese native culture. Mod. Finance Econ. 42, 3–19. 10.19559/j.cnki.12-1387.2022.04.001

[B86] Wang H. Yu J. Cui Z. (2024). Why go ahead in the face of danger? The impact of management openness on deviant innovation. J. Northeast. Univ. 26, 27–36. 10.15936/j.cnki.1008-3758.2024.02.004

[B87] Wang S. Noe R. A. Wang Z.-M. (2014). Motivating knowledge sharing in knowledge management systems: a quasi-field experiment. J. Manage. 40, 978–1009. 10.1177/0149206311412192

[B88] Xiao X. H. Liu W. X. Wang X. D.. (2018). Research on the impact of abusive supervision on employee knowledge sharing. Sci. Res. Manage. 39, 117–124 (in Chinese).

[B89] Xu J. Wei W. (2023). A theoretical review on the role of knowledge sharing and intellectual capital in employees' innovative behaviors at work. Heliyon 9:e20256. 10.1016/j.heliyon.2023.e20256 37767517 PMC10520787

[B90] Zhu J. Lin F. Zhang Y. Wang S. Tao W. Zhang Z. (2022). Exploring the effect of perceived overqualification on knowledge hiding: the role of psychological capital and person–organization fit. Front. Psychol. 13955661. 10.3389/fpsyg.2022.955661 36059786 PMC9435588

[B91] Zhu L. Liu C. Chen C. (2023). The collaborative symbiosis framework in the digital age: evolution of system efficiency and value. Chin. J. Manag. 20, 789–799.

